# Supplementary material for: Progress of the COVID-19: Persistence, Effectiveness, and Immune Escape of the Neutralizing Antibody in Convalescent Serum
Source: Pathogens. 2022 Dec 13;11(12):1531. doi: 10.3390/pathogens11121531 (PMC9782332; doi:10.3390/pathogens11121531)
Supplement: Supplementary file 1 [file pathogens-11-01531-s001.zip › pathogens-2032200-supplementary.pdf]

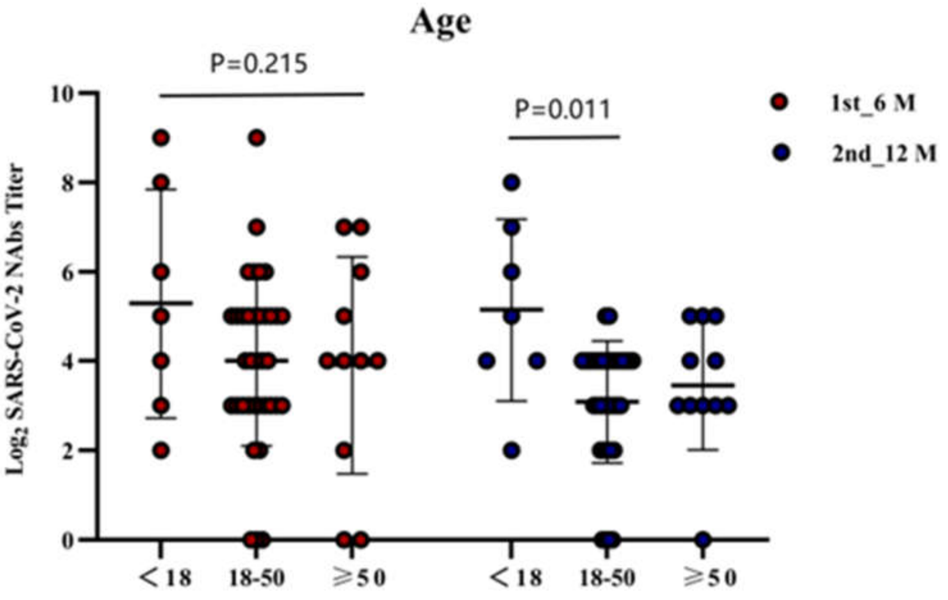

Figure S1. NAbs titers distribution of different ages at two intervals. NAbs titers were $\log_2$ processed. ***P*** values were determined with a two-tailed One-way ANOVA. NAbs: neutralizing antibodies; ANOVA: analysis of variance.

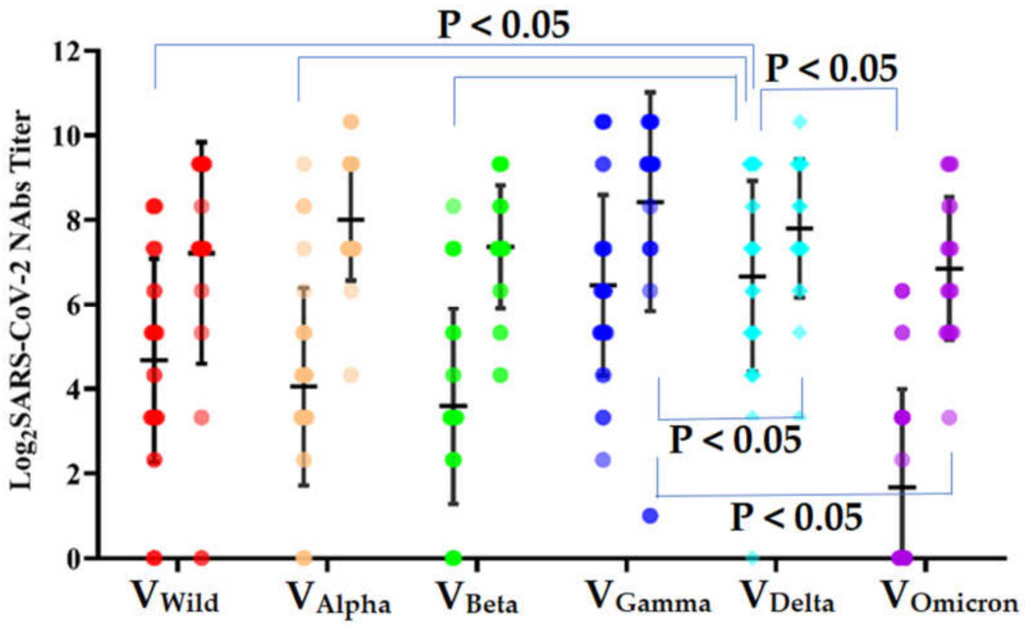

Figure S2. The NAbs titers in the cross-reactive test. The first and second columns of each horizontal coordinate point presented S_{Delta} and $S_{Omicron}$, respectively. NAbs titers were $\log_2$ processed. ***p*** values were determined with a student's *t*-test. NAbs: neutralizing antibodies.
